# Associations between Maternal Iron Supplementation in Pregnancy and Changes in Offspring Size at Birth Reflect Those of Multiple Micronutrient Supplementation

**DOI:** 10.3390/nu13072480

**Published:** 2021-07-20

**Authors:** Clive J. Petry, Ken K. Ong, Ieuan A. Hughes, David B. Dunger

**Affiliations:** 1 Department of Paediatrics, University of Cambridge, Cambridge CB2 0QQ, UK; Ken.Ong@mrc-epid.cam.ac.uk (K.K.O.); iah1000@cam.ac.uk (I.A.H.); dbd25@cam.ac.uk (D.B.D.); 2 MRC Department of Epidemiology, University of Cambridge, Cambridge CB2 0SL, UK; 3 Institute of Metabolic Science, University of Cambridge, Cambridge CB2 0QQ, UK

**Keywords:** fetal growth, gestational diabetes, development, adiposity, minerals, vitamins

## Abstract

It was previously observed that in a population of a high-income country, dietary multiple micronutrient supplementation in pregnancy was associated with an increased risk of gestational diabetes (GDM) and increased offspring size at birth. In this follow-up study, we investigated whether similar changes are observed with dietary iron supplementation. For this we used the prospective Cambridge Baby Growth Study with records of maternal GDM status, nutrient supplementation, and extensive offspring birth size measurements. Maternal iron supplementation in pregnancy was associated with GDM development (risk ratio 1.67 (1.01–2.77), *p* = 0.048, *n* = 677) as well as offspring size and adiposity (*n* = 844–868) at birth in terms of weight (β’ = 0.078 (0.024–0.133); *p* = 0.005), head circumference (β’ = 0.060 (0.012–0.107); *p* = 0.02), body mass index (β’ = 0.067 (0.014–0.119); *p* = 0.01), and various skinfold thicknesses (β’ = 0.067–0.094; *p* = 0.03–0.003). In a subset of participants for whom GDM statuses were available, all these associations were attenuated by adjusting for GDM. Iron supplementation also attenuated the associations between multiple micronutrient supplementation and these same measures. These results suggest that iron supplementation may mediate the effects associated with multiple micronutrient supplementation in pregnancy in a high-income country, possibly through the increased risk of developing GDM.

## 1. Introduction

Although evidence from middle and low-income countries suggests that dietary supplementation with multiple micronutrients in pregnancy is beneficial in terms of reducing the prevalence of preterm births and low birth weight babies, as well as the numbers born small for their gestational age (SGA) [1], its value in high-income countries is more controversial. Our recent observational study of multiple micronutrient supplementation in pregnancy also found positive associations even in a high-income country setting including increased offspring weight, head circumference, and skinfold thicknesses at birth [2]. Multiple micronutrient supplementation was also associated with increased maternal gestational diabetes (GDM) risk that may underpin the associations with increased offspring size at birth. At present, it is not known which individual or group of micronutrients caused the effects associated with multiple micronutrient supplementation in this study. It does not appear to be folic acid supplementation, however, as our assessment of effects in pregnancy produced results that failed to reflect those of supplementation with multiple micronutrients [3]. Another candidate micronutrient for underpinning associations with multiple micronutrient supplementation in pregnancy is iron.

Iron supplementation in pregnancy is common and indeed recommended in many countries [4]. In the UK, however, it is only recommended for women who have iron deficiency or iron deficiency anaemia [5,6]. Most such supplementation in pregnancy is therefore prophylactic and often taken with other vitamins and minerals in the form of multiple micronutrient preparations. It has been assumed that for iron replete women this is harmless [7] and that universal supplementation is beneficial in order to treat any developing cryptic iron deficiency or iron deficiency anaemia. In recent years, however, evidence has emerged of such dietary supplementation being associated with negative consequences [8,9,10,11,12,13]. Thus, systematic reviews and meta-analyses have demonstrated that both iron supplementation and having higher iron levels (either in the circulation or as biomarkers of its storage) are associated with an increased threat of developing GDM [8,9,10,11] and observational studies have demonstrated it to be associated with increased risk of pre-eclampsia [12,13], possibly mediated through increased oxidative stress. Dietary iron supplementation in pregnancy has also been reported to lead to increased offspring birth weight [14] and it has been speculated that there may also be long-term consequences for the children of pregnancies where iron was supplemented [15].

Following the previous studies linking dietary iron supplementation with GDM development [8,9,10,11] and the recent observation of increased GDM risk in women supplementing their diets with (iron-containing) multiple micronutrients in pregnancy [2], we hypothesised that iron supplementation in pregnancy mediated the previously observed associations between multiple micronutrient supplementation in (1) pregnancy and GDM risk and (2) offspring size at birth and adiposity. Furthermore, we postulated that we would observe similar associations with iron supplementation to those that were observed with multiple micronutrient supplementation [2]. We also assessed whether dietary iron supplementation in pregnancy was associated with an increased risk of developing an adverse hypertensive condition of pregnancy. We tested our hypothesis using a large normal birth cohort where mothers were prospectively tracked through pregnancy until the birth of their baby and detailed measures of the size at birth of the offspring were recorded [16].

## 2. Materials and Methods

### 2.1. Cambridge Baby Growth Study

The prospective longitudinal Cambridge Baby Growth Study (CBGS) recruited women from early pregnancy ultrasound clinics at the Rosie Maternity Hospital, Cambridge, UK, to the first phase between 2001 and 2009 [16]. A total of 2229 pregnant women over the age of 16 years were recruited to this part of the study, although 571 of them withdrew before their baby was born and thus self-excluded. Women with multifetal pregnancies were also excluded from the present analysis (due to the considerable effect on offspring birth size [17]), as were women who did not fill in and return their pregnancy questionnaires. The preponderance of the offspring in this cohort (95.3%) were White, with fewer offspring being of mixed race (1.7%), Black African or Caribbean (1.3%), and Asian (1.7%).

Around week 28 of pregnancy, 1074 of the women underwent oral glucose tolerance testing (OGTTs) using 75 g glucose loads consumed orally after overnight fasting [17]. The results from the OGTTs were used to classify GDM using diagnostic criteria suggested by the International Association of Diabetes in Pregnancy Study Group [18]. The prospective data from this analysis was supplemented with blood pressure-related data collected retrospectively to obtain as many useful records from individual pregnancies as possible. Pre-eclampsia was classified from hospital notes using the terms “preeclampsia”, “pre-eclampsia”, “pre-eclamptic toxaemia”, and “PET”. Gestational hypertension was classified in 720 of all the women in the cohort using a combination of blood pressure measurements taken after week 20 of pregnancy that were recorded from the hospital notes [19] and a hospital diagnosis of “gestational hypertension”, “pregnancy-induced hypertension”, or “PIH” [19]. Records of blood pressures in the other women were missing due to either hospital notes not being available to us or those hospital notes not containing the relevant results. Low birth weight was defined using the standard criterion of an unadjusted birth weight of less than 2.5 kg. SGA was classified as a birth weight that was less than the 10th percentile for gestational age when compared with UK growth charts. A birth was described as premature if it occurred at less than 37 weeks gestation. The body mass index (BMI) was calculated as the body weight (pre-pregnancy for the mother or at birth for the baby) divided by the height or length squared. The ponderal index was calculated as the BMI divided by the length. Pregnancy weight gain was determined as the pregnancy weight (in the final week of pregnancy) minus the pre-pregnancy weight, both of which were self-reported in the pregnancy questionnaire. The index of multiple deprivation was collected from published tables derived using the participants’ residential postcodes [2].

### 2.2. Ethical Review

Ethical approval for the CBGS (00/325) was granted by the Cambridge Local Research Ethics Committee, Addenbrooke’s Hospital, Cambridge, UK. Women gave informed consent for both themselves and their babies. All procedures were followed according to the institutional guidelines.

### 2.3. Assessment of Iron Supplementation in Pregnancy

At recruitment, an extensive questionnaire was given to each of the participants. They were asked to complete it as the pregnancy progressed (with assistance from trained research nurses if required) [2,17]. The questionnaires were collected post-partum. One of the lifestyle questions asked included, “Have you taken any dietary supplements during the pregnancy?” If this was answered “yes”, there was a table to fill in with “Name of Product”, “Frequency”, and “Gestational Weeks” as column headings. Some of the rows of the table were pre-filled to allow ticking; one of these was specifically for iron supplements. Although 1239 of the CBGS participants filled in their pregnancy questionnaires, only 1001 participants gave unambiguous responses to the question about dietary supplementation and thus the remaining 238 participants were excluded from the present analysis. Where a brand of dietary supplements was listed (rather than the specific variety of vitamins/minerals), an internet search was used to find whether the supplements included iron [2]. Where a brand was listed without the name of the variety used, an assumption was made that the supplement that was taken was that brand’s biggest-selling variety of pregnancy supplement. Iron supplementation due to maternal anaemia as identified as any disease indication was also self-reported. Multiple micronutrient supplementation was categorised in the same manner as iron [2].

### 2.4. Assessment of Offspring Size at Birth

Birth weights were collected from hospital notes. Other new-born measurements were made as close to birth as possible (at a median (interquartile range) of 2–16 days) by trained paediatric nurses. These measurements included the baby’s length, head circumference, and skinfold thickness at four sites. They were all measured in triplicates and the mean was used in the analyses. Details of how the measurements were made have been presented previously [2].

### 2.5. Assessment of the Potential Confounding Effects of Food Frequency Intakes on GDM Risk

A simple but validated food frequency questionnaire to be filled in as the pregnancy progressed was also included in the full pregnancy questionnaire. Specific food intakes were converted into a frequency of consumption score as previously described [17]. These were then used to investigate if they confounded the link between iron supplementation in pregnancy and increased GDM risk.

### 2.6. Statistical Analysis

This analysis follows a regrouping of data from pregnant women who were participants in the CBGS that were included in the previous multiple micronutrient and folic acid analyses [2,3]. The exposed group contained data from women who supplemented their diets with iron alone, with iron and folic acid alone (or other single micronutrient), or with multiple micronutrients that included iron. The non-exposed group contained data from women who supplemented their diets with micronutrients alone that did not contain iron (e.g., folic acid, vitamin C, or vitamin D) or who did not supplement their diets with micronutrients. Similar to the previous studies [2,3], it was analysed comparing data from these two groups. Casewise deletion was generally used to address missing data unless specified otherwise. Linear regression (adjusting for confounders where necessary) was used to analyse continuous variables. If residuals were skewed, data transformation (e.g., logarithmic adjustment) was applied prior to analysis. Logistic regression (reporting relative risks (RR) via the use of log binominal regression), Fisher’s exact, and χ2-tests were used to analyse categorical variables as appropriate. The statistical analyses were conducted using Stata (version 13.1; Stata Corp., from Timberlake Consultants Ltd., Richmond, Surrey, UK) or R (version 3.6.1; The R Foundation for Statistical Computing, Vienna, Austria), and *p* < 0.05 was used to define statistical significance.

Sensitivity analyses were performed by repeating the association analyses in women who supplemented their diets with iron alone (rather than via multiple micronutrient supplementation) and by excluding those women who reported a diagnosis of anaemia in pregnancy. Effects of potential mediators that may underpin the significant associations (GDM and weight gain in pregnancy) were tested in women for whom these data were available by examining the effect of adjusting for those potential mediators on associations between iron supplementation and measures of size at birth. Similarly, iron supplementation was tested as a mediator of the significant associations between (1) multiple micronutrient supplementation and (2) maternal GDM risk, offspring birth size, and adiposity.

## 3. Results

### 3.1. Study Participants

There were few clinical disparities between the women whose data was included in this analysis and those that were excluded from it (Table S1). Those that were evident were of the presence of a multifetal pregnancy (as data from these mothers were specifically excluded from the analysis) and a lower proportion that smoked in a cohort where smoking in pregnancy was relatively uncommon [16].

Most clinical characteristics did not differ between those women who took iron-containing supplements in pregnancy (*n* = 582; 60.1%) and those that did not (*n* = 387; 39.9%) (Table 1). Of those that did, there was a slightly higher proportion of first pregnancies in the women that supplemented their diets with iron. In addition, proportionally more women (*n* = 524) amongst those that classified as taking iron took multiple micronutrient supplements. In fact, as all the multiple micronutrient preparations used contained iron, participants who supplemented with multiple micronutrients were all classified as taking iron (apart from those that were excluded from the whole analysis due to having multifetal pregnancies). All the multiple micronutrient preparations also contained folic acid, supported by the fact that there was a higher proportion of those that took iron supplements than those that did not also supplement with folic acid. All but one of the women who reported experiencing anaemia during pregnancy (*n* = 27) took iron supplements (as single supplements; the one participant with anaemia who did not use iron supplements took folic acid). Although there were a wide range of start times, the modal time of when women started supplementing with iron was around conception (Figure S1) and many of the women continued supplementing throughout pregnancy.

### 3.2. Associations with Adverse Outcomes of Pregnancy and Offspring Size at Birth

A higher proportion of women who took iron supplements developed GDM than the proportion of those that did not (RR 1.67 (1.01–2.77); *n* = 677; *p* = 0.048; Table 2). This raised RR was only partially attenuated by adjusting for various food frequency intakes (Table S2). There was a borderline increase in weight gain in those women who supplemented their diets with iron (*p* = 0.05). There were no differences between iron supplementation groups in the proportion of women that developed either gestational hypertension or pre-eclampsia, or in the proportion that gave birth to premature, SGA, or low birth weight babies.

Maternal iron supplementation in pregnancy was associated with increased offspring weight (*p* = 0.005), head circumference (*p* = 0.02), BMI (*p* = 0.01), and skinfold thicknesses in three of the four sites (*p* = 0.03–0.003) (the fourth having a borderline significance of *p* = 0.05) (Table 3). In the participants who supplemented with iron that was not part of a multiple micronutrient preparation (*n* = 60–63, around 12% of the total number of participants that supplemented their diets with iron), the maternal iron supplementation was still associated with increased offspring subscapular skinfold thicknesses (*p* = 0.03; Table S3).

### 3.3. Exploration of Potential Effects Related to Anaemia, GDM, and Pregnancy Weight Gain

Excluding women who reported being anaemic during pregnancy, associations persisted between maternal iron supplementation in pregnancy and both the development of GDM (RR 1.70 (1.01–2.86); *p* = 0.045) and offspring size at birth (Table S4). In 663 women for whom GDM statuses were available and who were included in both the multiple micronutrient and iron supplementation analyses, as expected, multiple micronutrient supplementation was associated with GDM (RR 1.87 (1.13–3.08); *p* = 0.02). When adjusted for iron supplementation, the significance of the association with multiple micronutrient supplementation in the same women was lost (RR 1.76 (0.64–4.81); *p* = 0.3). Adjusting for iron supplementation also attenuated associations between dietary supplementation with multiple micronutrients and offspring size at birth (Table 4).

In 608 women for whom relevant data were available, adjusting for GDM attenuated the various associations between maternal iron supplementation in pregnancy and offspring size at birth (Table S5). A smaller effect was noted in 632 women with relevant data when adjusting the statistical models for pregnancy weight gain (Table S6).

## 4. Discussion

In this analysis, women who supplemented their diets with iron in pregnancy had an increased risk of developing GDM as well as increases in various measures of offspring size at birth (including their weight, head circumference, BMI, and skinfold thicknesses). These findings are very similar to previously observed associations with pregnant women who supplemented their diets with multiple micronutrients [2]. Unlike with the lack of effects associated with folic acid supplementation in pregnancy [3], these results suggest that the multiple micronutrient supplementation associations may have been mediated at least in part by iron supplementation. In the UK, iron supplementation is not routinely recommended for pregnant women until after either iron deficiency or iron deficiency anaemia has been diagnosed [20]. However, in the CBGS it was common to supplement maternal diets with iron, most often in the form of multivitamin and mineral tablets (90.6% of those that supplemented their diets with iron). The format used in this study results in a difficulty to differentiate the effects of multiple micronutrient supplementation from those of iron supplementation as all the multiple micronutrient preparations contained iron. The main difference from the multiple micronutrient study [2] relates to the data collected from the women who supplemented their diets with iron but did not take multiple micronutrients. These would have been considered non-exposed in the previous study [2]; thus, in comparing results from both studies, the key associations from the original study would have been expected to be somewhat attenuated by their inclusion in the exposed group if iron were not involved in mediating them. This was not what was observed, however. In addition, when assessing associations with offspring size at birth in the small subset of women who supplemented their diets with iron alone (along with non-exposed participants), the vast majority of the associations were in the same direction as those in the full study, albeit mainly non-significantly probably due to the reduction in statistical power. The association with offspring subscapular skinfold thicknesses with maternal iron supplementation in pregnancy was still significant, however. These results are therefore consistent with a role for maternal iron supplementation in mediating the main associations from the present study. In support of this are findings from clinical trials where iron was supplemented in isolation [21,22]. Consistent with a role for iron in mediating the multiple micronutrient associations, in the present analysis iron supplementation attenuated previously observed associations between multiple micronutrient supplementation and both the maternal risk of developing GDM and increases in offspring birth size [2].

In addition to results suggesting that iron supplementation may have mediated previously observed associations with multiple micronutrient supplementation [2], this analysis confirmed previous studies that found associations between iron supplementation in pregnancy and an increased risk of the development of GDM [8,9,10,11] (by around 70% in the present analysis). It also confirmed previous observations linking maternal iron supplementation in pregnancy with increased offspring birth weight [14], extending this finding to positive associations with various other measures of offspring size at birth such as BMI and three of the four skinfold measurements that were assessed (the other one reaching borderline significance in the same direction). This suggests that iron supplementation in pregnancy is associated with increased offspring adiposity at birth. Adiposity is thought to be better estimated by ponderal index than BMI at birth [23] but the association with ponderal index in the present analysis was in the same direction as that of BMI even if statistical significance was not reached. We did not find an effect on length at birth which is consistent with a meta-analysis of randomised trials and cohort studies regarding the effect of iron supplementation in pregnancy [24]. Neither could we find links between adverse pregnancy outcomes such as premature birth, pre-eclampsia and gestational hypertension, and maternal dietary iron supplementation in pregnancy. However, these analyses could have been somewhat underpowered in the CBGS population where these adverse outcomes appeared to occur less frequently than GDM. The lack of association between maternal iron supplementation in pregnancy and maternal adverse hypertensive pregnancy outcomes does not indicate that there will not be long-term changes in the blood pressure of the offspring [25].

We found that GDM attenuated all the associations between iron supplementation in pregnancy and the various measures of size at birth. These assessments were conducted at a time when there was a temporal trend for a rising prevalence of GDM in this population [26], although the relationship between iron supplementation in pregnancy and the development of GDM was not so strong that there was a concurrent temporal trend in dietary iron supplementation (data not shown). The results from the present study suggest that GDM may mediate at least part of the link between maternal dietary iron supplementation in pregnancy and increased offspring adiposity and size at birth. Consistent with this, CBGS participants who developed GDM tended to give birth to heavier babies [27]. It has been suggested that adverse outcomes of pregnancy may be linked to iron supplementation through increases in oxidative stress [28]. In reviewing the links between iron status, oxidative stress, and GDM, Zein et al. [29] suggested a mechanism whereby dietary iron supplementation may initially lead to a state of increased oxidative stress (GDM being of such a state [29,30]). This increased oxidative stress may then enhance insulin resistance (possibly resulting from a reduced ability of the liver to extract insulin). The increased oxidative stress may also even lead to pancreatic β-cell dysfunction. The combination of the enhanced insulin resistance and the pancreatic β-cell dysfunction in turn may then contribute to the development of GDM [29].

The strengths of this study include the levels of detail available to us in relation to the assessment of size at birth and maternal GDM status in a relatively large number of newborns and women that were part of a well-characterised cohort. It also has a number of limitations, however. Firstly and most importantly, we analysed iron supplementation in pregnancy only as a dichotomous exposure; thus, there was no account of the stage of pregnancy when iron was supplemented, the doses of iron consumed, the dietary iron consumption, or maternal iron status. However, this approach had the advantage of increasing the numbers of participants available to us due to fewer exclusions resulting from missing iron-related data. Also, it has been shown by meta-analysis that the duration of iron use in pregnancy is not significantly associated with outcomes such as increases in birth weight [24]. A second limitation is that data relating to iron supplementation and anaemia were self-reported that could have introduced inaccuracies [31]. However, the questionnaire that we used was based on one that was validated by phone interviews [32] that may have limited inaccuracies. The overall extent to which data were missing is a further weakness. However, this is common in cohort studies that are used for multiple analyses. While missing data could have been imputed in the present analysis, this might have increased measurement errors and introduced inaccurate confidence interval estimates. Instead, casewise deletion of missing data was generally used.

In conclusion, this analysis confirmed previous reported associations between iron supplementation in pregnancy and both maternal GDM and increased offspring birth weight. We extended this to other measures of size at birth and demonstrated that the principal reason for the increased weight seems to be increased adiposity. These associations appeared to be mediated by the increased risk of GDM. The main associations with iron supplementation reflected and attenuated those with multiple micronutrient supplementation in this cohort, suggesting that it may be the main mediator of such associations.

## Figures and Tables

**Table 1:** A comparison of the characteristics of those CBGS maternal participants who supplemented their diets with iron during pregnancy and those that did not.

Maternal Characteristic	No Iron Supplementation (*n* = 252–387)	Iron Supplementation (*n* = 377–582)	*p*-Value
Age (years)	33.2 (32.8–33.6)	33.6 (33.3–34.0)	0.1
Height (m)	1.66 (1.65–1.66)	1.66 (1.66–1.67)	0.2
Pre-pregnancy weight (kg)	66.3 (64.9–67.6)	66.2 (65.0–67.3)	0.9
Pre-pregnancy BMI (kg/m^2^)	24.2 (23.7–24.7)	24.0 (23.6–24.3)	0.5
Weight gain in pregnancy (kg)	7.8 (7.0–8.7)	8.9 (8.2–9.5)	0.05
Index of multiple deprivation	8.7 (8.2–9.2)	8.7 (8.3–9.1)	0.9
First pregnancy (*n* yes, *n* no)	148 yes, 238 no	277 yes, 304 no	0.03
Smoked during pregnancy (*n*)	14 yes, 372 no	17 yes, 564 no	0.5
Anaemia (*n*)	1 yes, 366 no	26 yes, 540 no	2.8 × 10^−5^
Supplemented with multiple (3 or more) micronutrients in pregnancy (*n*)	8 yes, 378 no	516 yes, 59 no	1.1 × 10^−157^
Supplemented with folic acid in pregnancy (*n*)	204 yes, 183 no	567 yes, 10 no	3.0 × 10^−67^
Length of pregnancy (weeks)	39.8 (39.6–40.0)	39.9 (39.8–40.1)	0.2
Birth presentation (*n* head, *n* breach, and *n* other)	304 head, 16 breach, and 30 other	433 head, 20 breach, and 51 other	0.7
Delivery (*n* vaginal, *n* vacuum, *n* forceps, *n* elective section, and *n* acute section)	231 vaginal, 20 vacuum, 16 forceps, 61 elective section, and 55 acute section	333 vaginal, 40 vacuum, 31 forceps, 78 elective section, and 84 acute section	0.6

Data are mean (95% confidence interval) or number of participants. *p*-values were gained using linear regression, χ^2^-test, or Fisher’s exact tests as appropriate.

**Table 2:** Numbers and relative risks of various adverse conditions of pregnancy according to whether or not the participants supplemented their diets with iron.

Condition	No Iron Supplementation	Iron Supplementation	Relative Risk	*p*-Value
Gestational diabetes (*n* yes, *n* no)	19 yes, 247 no	49 yes, 362 no	1.669(1.006–2.770)	0.048
Pre-eclampsia (*n* yes, *n* no)	7 yes, 385 no	8 yes, 585 no	0.755(0.276–2.067)	0.6
Gestational hypertension (*n* yes, *n* no)	11 yes, 175 no	16 yes, 272 no	0.939(0.446–1.979)	0.9
Low birth weight (*n* yes, *n* no)	12 yes, 373 no	15 yes, 566 no	0.828(0.392–1.750)	0.6
Small for gestational age (*n* yes, *n* no)	2 yes, 383 no	3 yes, 578 no	0.994 (0.167–5.921)	1.0
Premature birth (*n* yes, *n* no)	7 yes, 379 no	11 yes, 571 no	1.042 (0.408–2.665)	0.9

Data are number of participants or mean (95% confidence interval). *p*-values were gained using logistic regression.

**Table 3:** Associations between iron supplementation status in pregnancy and indices of offspring size at birth.

Measure	No Maternal Iron Supplementation in Pregnancy	Maternal Iron Supplementation in Pregnancy	Standardised β	*p*-Value
Weight (kg)	3.443 (3.396–3.489) (*n* = 345)	3.529 (3.491–3.566) (*n* = 523)	0.078 (0.024–0.133)	0.005
Length * (cm)	51.3 (51.1–51.5) (*n* = 332)	51.6 (51.4–51.7) (*n* = 514)	0.041 (−0.008–0.089)	0.1
Head circumference * (cm)	35.2 (35.1–35.3) (*n* = 332)	35.4 (35.3–35.5) (*n* = 515)	0.055 (0.009–0.100)	0.02
BMI * (kg/m^2^)	13.0 (12.9–13.2) (*n* = 331)	13.2 (13.1–13.3) (*n* = 513)	0.067 (0.014–0.119)	0.01
Ponderal index * (kg/m^3^)	25.4 (25.1–25.7) (*n* = 331)	25.7 (25.5–26.0) (*n* = 513)	0.049 (−0.002–0.100)	0.06
Flank skinfold thickness * (mm)	6.0 (5.8–6.1) (*n* = 333)	6.2 (6.1–6.3) (*n* = 513)	0.067 (0.006–0.127)	0.03
Quadriceps skinfold thickness * (mm)	7.8 (7.5–8.0) (*n* = 332)	8.0 (7.9–8.2) (*n* = 514)	0.053 (0–0.105)	0.05
Subscapular skinfold thickness * (mm)	5.2 (5.1–5.4) (*n* = 333)	5.5 (5.4–5.6) (*n* = 513)	0.086 (0.027–0.146)	0.005
Triceps skinfold thickness * (mm)	5.3 (5.2–5.5) (*n* = 333)	5.6 (5.5–5.7) (*n* = 513)	0.094 (0.033–0.156)	0.003

Standardised βs are shown as mean (95% confidence interval). *p*-values were gained using multiple linear regression models. All models were adjusted for gestational age at birth, parity (as a continuous variable), smoking during pregnancy, offspring sex, and maternal pre-pregnancy BMI. * Models additionally adjusted for age at assessment.

**Table 4:** Subgroup comparison assessing the confounding effect of iron supplementation on the associations between multiple micronutrient supplementation in pregnancy and indices of size at birth and adiposity.

Measure	*N*	Association with Multiple Micronutrient Supplementation		Association with Multiple Micronutrient Supplementation Adjusted for Iron Supplementation	
		Standardised β	*p*-Value	Standardised β	*p*-Value
Weight	849	0.063 (0.008–0.118)	0.03	−0.036 (−0.142–0.070)	0.5
Length *	827	0.034 (−0.015–0.084)	0.2	−0.027 (−0.122–0.068)	0.6
Head circumference *	828	0.052 (0.003–0.101)	0.04	0.001 (−0.094–0.095)	1.0
BMI *	825	0.049 (−0.005–0.103)	0.08	−0.042 (−0.146–0.062)	0.4
Ponderal index *	825	0.036 (−0.016–0.088)	0.2	−0.025 (−0.125–0.076)	0.6
Flank skinfold thickness *	827	0.065 (0.003–0.127)	0.04	0.021 (−0.098–0.141)	0.7
Quadriceps skinfold thickness *	827	0.061 (0.007–0.115)	0.03	0.052 (−0.052–0.157)	0.3
Subscapular skinfold thickness *	827	0.070 (0.009–0.130)	0.03	−0.027 (−0.144–0.090)	0.7
Triceps skinfold thickness *	827	0.096 (0.034–0.159)	0.003	0.042 (−0.078–0.163)	0.5

Values in the table only include data from those pregnancies where the mothers were part of both the multiple micronutrient and iron studies. Standardised βs are shown as mean (95% confidence interval). *p*-values were gained using multiple linear regression models. All models were adjusted for gestational age at birth, parity (as a continuous variable), smoking during pregnancy, offspring sex, and maternal pre-pregnancy BMI. * Models additionally adjusted for age at assessment.

## Data Availability

The data presented in this study are openly available in the University of Cambridge Apollo repository at https://doi.org/10.17863/CAM.70019 (accessed on 19 July 2021).

## Supplementary Materials

The following are available online at https://www.mdpi.com/article/10.3390/nu13072480/s1: Figure S1: (a) The time, relative to the start of pregnancy, when iron supplementation was started in those women who did supplement, and (b) the total length of time that these women supplemented with iron; Table S1: comparison of characteristics of those that were included in this present analysis and those that were excluded from it; Table S2: effect of including specific food frequency intakes as confounders in the logistic regression models describing the relationship between iron supplementation during pregnancy and risk of GDM in the Cambridge Baby Growth Study; Table S3: associations between iron supplementation status in pregnancy and indices of offspring size at birth; Table S4: associations of iron supplementation in pregnancy with (a) outcomes of pregnancy and (b) offspring size at birth and adiposity in women and babies in absence of reported maternal anaemia; Table S5: subgroup comparison assessing the effect of maternal GDM on associations between iron supplementation in pregnancy and offspring size at birth; and Table S6: subgroup comparison assessing the effect of maternal weight gain in pregnancy on associations between iron supplementation in pregnancy and offspring size at birth.
