# Supplementary material for: Associations between Maternal Iron Supplementation in Pregnancy and Changes in Offspring Size at Birth Reflect Those of Multiple Micronutrient Supplementation
Source: Nutrients. 2021 Jul 20;13(7):2480. doi: 10.3390/nu13072480 (PMC8308651; doi:10.3390/nu13072480)
Supplement: Supplementary file 1 [file nutrients-13-02480-s001.zip › nutrients-1249629-supplementary.pdf]

Supplementary Figures and Tables for Petry et al., "Associations between Maternal Iron Supplementation in Pregnancy and Changes in Offspring Size at Birth Reflect those of Multiple Micronutrient Supplementation"

Figure S1 (a) The time, relative to the start of pregnancy, when iron supplementation was started in those women who did supplement, and (b) The total length of time that these women supplemented with iron.

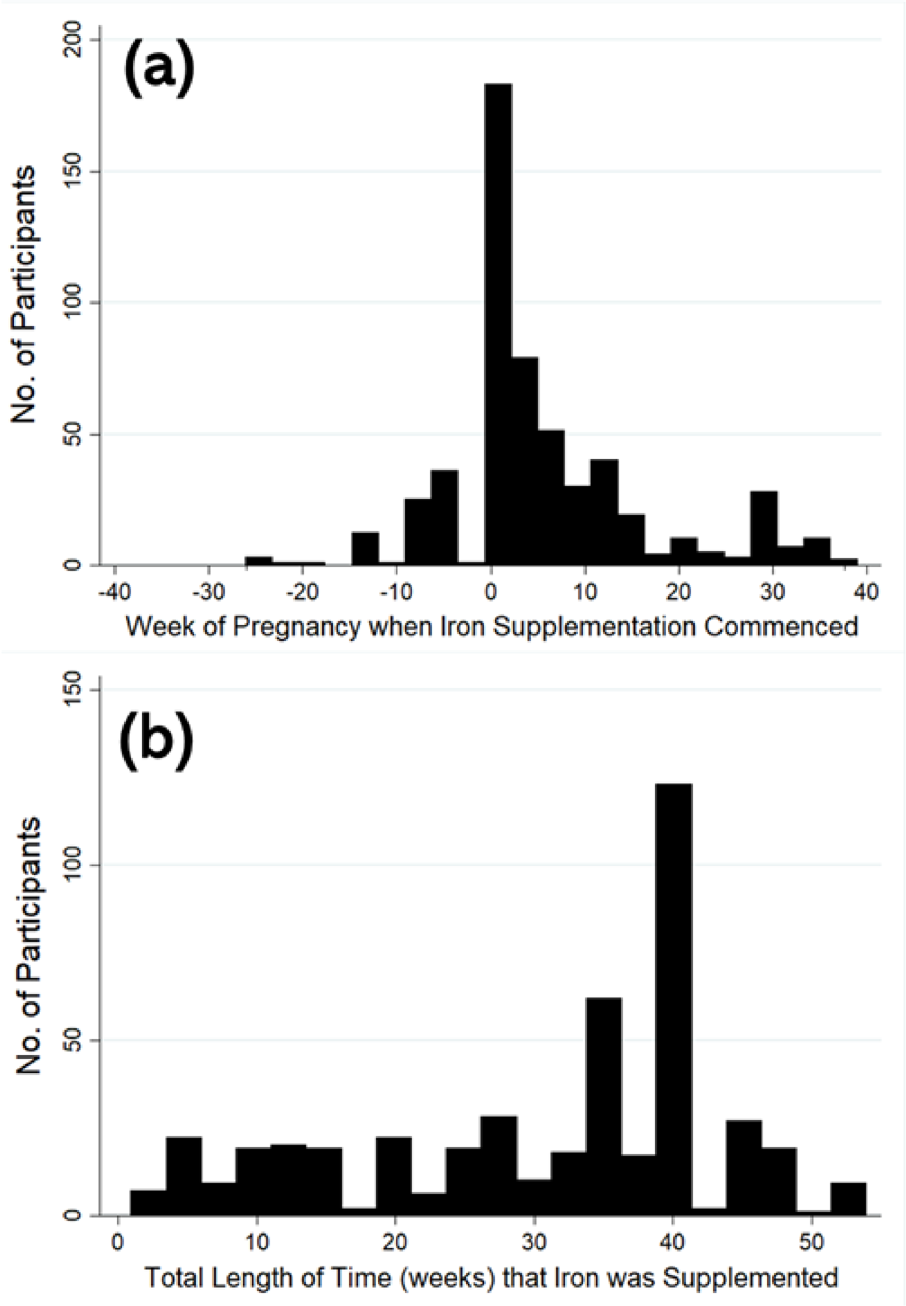

Table S1 Comparison of characteristics of those that were included in this present analysis and those that were excluded from it.

**Characteristic**	**Excluded from this analysis**	**Included in this analysis**	***p*-value**
Age (years)	33.6 (33.2—34.0)<br>(n=437)	33.5 (33.2—33.7)<br>(n=898)	0.5
Maternal pre-pregnancy BMI (kg/m^2)	24.2 (23.7—24.7)<br>(n=315)	24.0 (23.7—24.3)<br>(n=872)	0.6
Weight gain during pregnancy (kg)	7.5 (6.6—8.4)<br>(n=226)	8.5 (8.0—9.0)<br>(n=648)	0.06
Smoked during pregnancy (n)	55 yes, 663 no	31 yes, 936 no	1.5×10^{-5}
Gestational diabetes (n)	43 yes, 364 no	68 yes, 609 no	0.8
Gestational hypertension (n)	17 yes, 229 no	27 yes, 447 no	0.5
Preeclampsia (n)	7 yes, 681 no	15 yes, 970 no	0.3
Multifetal pregnancy (n)	50 twin, 647 singleton	0 twin, 985 singleton	1.2×10^{-16}
Gestational age at birth of offspring (weeks)*	39.7 (39.6—39.9)<br>(n=689)	39.8 (39.7—39.9)<br>(n=968)	0.2
Adjusted offspring birth weight (kg)**	3.484 (3.434—3.533)<br>(n=314)	3.473 (3.444—3.503)<br>(n=870)	0.7

Data are numbers of participants or mean (95% confidence interval)

*adjusted for twin pregnancies

**adjusted for gestational age at birth, parity, sex, smoking, maternal BMI and twin pregnancies

<u>Table S2</u> Effect of including specific food frequency intakes as confounders in the logistic regression models describing the relationship between iron supplementation during pregnancy and risk of GDM in the Cambridge Baby Growth Study.

**Food/Drink type**	**N**	**Relative Risk**	***p*-value**
None	677	1.67 (1.01—2.77)	0.048
Baked beans	671	1.68 (1.02—2.79)	0.044
Bean curd	668	1.68 (1.01—2.80)	0.046
Beer	647	1.81 (1.07—3.07)	0.03
Canned fish	671	1.67 (1.01—2.78)	0.047
Canned meat	670	1.67 (1.00—2.76)	0.049
Canned (non-baked) beans	668	1.57 (0.94—2.61)	0.09
Chocolate	668	1.63 (0.98—2.71)	0.06
Cocoa	646	1.77 (1.04—3.01)	0.04
Coffee	663	1.72 (1.02—2.88)	0.04
Cola	650	1.71 (1.01—2.91)	0.047
Dried fruit	669	1.73 (1.04—2.87)	0.04
Eggs	672	1.67 (1.00—2.77)	0.049
Fish	669	1.67 (1.01—2.77)	0.046
Fresh fruit	672	1.65 (0.99—2.74)	0.05
Fresh fruit juice	667	1.66 (1.00—2.76)	0.05
Fresh green vegetables	669	1.64 (0.99—2.72)	0.06
Fresh non-green vegetables	670	1.66 (1.00—2.75)	0.05
Frozen vegetables	670	1.66 (1.00—2.76)	0.049
Hard cheese	672	1.66 (1.00—2.75)	0.05
Liver	671	1.63 (0.99—2.71)	0.06

Meat	671	1.67 (1.00—2.77)	0.049
Organic food	668	1.68 (1.01—2.81)	0.047
Poultry	670	1.66 (1.00—2.75)	0.05
Pulses	670	1.61 (0.97—2.69)	0.07
Salad	662	1.62 (0.98—2.70)	0.06
Shellfish	664	1.62 (0.97—2.69)	0.06
Soft cheese	655	1.63 (0.98—2.72)	0.06
Soya	669	1.61 (0.97—2.69)	0.07
Spirits	647	1.64 (0.97—2.77)	0.06
Tea	659	1.74 (1.02—2.96)	0.04
Tinned fruit	669	1.67 (1.01—2.77)	0.048
Tinned vegetables	672	1.69 (1.02—2.81)	0.04
White fish	667	1.64 (0.99—2.72)	0.06
Wine	654	1.75 (1.03—2.97)	0.04
Yogurt	669	1.63 (0.98—2.71)	0.06

Data are either individual numbers or means (95% confidence intervals).

Table S3 Associations between iron supplementation status in pregnancy and indices of offspring size at birth.

Measure	No Maternal Iron Supplementation in Pregnancy	Maternal Iron Supplementation (not Multiple Micronutrients) in Pregnancy	Standardized β	p-value
Weight (kg)	3.443 (3.396—3.489) (n=345)	3.523 (3.413—3.633) (n=63)	0.053 (-0.026—0.133)	0.2
Length* (cm)	51.3 (51.1—51.5) (n=332)	51.7 (51.3—52.2) (n=61)	0.041 (-0.025—0.107)	0.2
Head circumference* (cm)	35.2 (35.1—35.3) (n=332)	35.4 (35.1—35.7) (n=61)	0.030 (-0.037—0.096)	0.4
BMI* (kg/m^2)	13.0 (12.9—13.2) (n=331)	13.2 (12.8—13.5) (n=60)	0.044 (-0.035—0.123)	0.3
Ponderal index* (kg/m^3)	25.4 (25.1—25.7) (n=331)	25.5 (24.8—26.1) (n=60)	0.022 (-0.52—0.096)	0.6
Flank skinfold thickness* (mm)	6.0 (5.8—6.1) (n=333)	6.2 (5.8—6.7) (n=61)	0.040 (-0.054—0.133)	0.4
Quadriceps skinfold thickness* (mm)	7.8 (7.5—8.0) (n=332)	7.9 (7.3—8.4) (n=61)	-0.003 (-0.084—0.078)	0.9
Subscapular skinfold thickness* (mm)	5.2 (5.1—5.4) (n=333)	5.6 (5.3—6.0) (n=61)	0.097 (0.008—0.186)	0.03
Triceps skinfold thickness* (mm)	5.3 (5.2—5.5) (n=333)	5.5 (5.2—5.8) (n=61)	0.023 (-0.066—0.113)	0.6

Standardized βs are presented as means (95% confidence intervals). All models adjusted for gestational age at birth, parity, smoking during pregnancy, offspring sex and maternal pre-pregnancy BMI. *Models additionally adjusted for age at assessment.

Table S4 Associations of iron supplementation in pregnancy with (a) outcomes of pregnancy and (b) offspring size at birth and adiposity, in women and babies without reported maternal anaemia.

(a)

Pregnancy Outcome	N	Risk Ratio	p-value
Gestational diabetes	626	1.70 (1.01—2.86)	0.045
Pre-eclampsia	906	0.78 (0.28—2.12)	0.6
Gestational hypertension	450	0.98 (0.45—2.14)	1.0
Low birth weight	895	0.80 (0.36—1.77)	0.6
SGA	895	2.04 (0.21—19.50)	0.5
Premature birth	897	0.87 (0.33—2.33)	0.8

(b)

Measure	No Maternal Iron Supplementation in Pregnancy	Maternal Iron Supplementation in Pregnancy	Standardized β	p-value
Weight (kg)	3.436 (3.389—3.484) (n=327)	3.520 (3.481—3.559) (n=481)	0.075 (0.019—0.131)	0.008
Length* (cm)	51.3 (51.1—51.5) (n=315)	51.5 (51.3—51.7) (n=473)	0.045 (-0.006—0.095)	0.08
Head circumference*	35.2 (35.0—35.3) (n=315)	35.4 (35.2—35.5) (n=473)	0.053 (0.003—0.102)	0.04
BMI* (kg/m^2)	13.0 (12.9—13.2) (n=314)	13.2 (13.1—13.3) (n=472)	0.059 (0.005—0.113)	0.03
Ponderal index* (kg/m^3)	25.5 (25.2—25.7) (n=314)	25.7 (25.5—26.0) (n=472)	0.040 (-0.012—0.093)	0.1
Flank skinfold thickness* (mm)	6.0 (5.8—6.1)	6.2 (6.0—6.3)	0.062 (-0.001—0.125)	0.05

	(n=316)	(n=472)		
Quadriceps skinfold thickness* (mm)	7.8 (7.5—8.0) (n=315)	8.0 (7.8—8.2) (n=473)	0.046 (-0.009—0.101)	0.1
Subscapular skinfold thickness* (mm)	5.2 (5.1—5.3) (n=316)	5.4 (5.3—5.6) (n=472)	0.084 (0.022—0.146)	0.008
Triceps skinfold thickness* (mm)	5.3 (5.2—5.5) (n=316)	5.6 (5.5—5.7) (n=472)	0.091 (0.028—0.154)	0.005

Data are mean (95% confidence interval) or number of participants.

All models in (b) adjusted for gestational age at birth, parity, smoking during pregnancy, offspring sex and maternal pre-pregnancy BMI. *Models additionally adjusted for age at assessment.

Table S5 Subgroup comparison assessing the effect of maternal GDM on associations between iron supplementation in pregnancy and offspring size at birth.

**Measure**	**N**	**Unadjusted for GDM**		**Adjusted for GDM**	
		**Standardized β**	**p-value**	**Standardized β**	**p-value**
Weight	608	0.072 (0.007—0.137)	0.03	0.058 (-0.006—0.123)	0.08
Length*	591	0.029 (-0.029—0.087)	0.3	0.022 (-0.036—0.080)	0.5
Head Circumference*	592	0.042 (-0.016—0.101)	0.2	0.043 (-0.015—0.102)	0.1
BMI*	590	0.063 (-0.001—0.127)	0.05	0.054 (-0.010—0.118)	0.1
Ponderal Index*	590	0.051 (-0.011—0.112)	0.1	0.045 (-0.017—0.107)	0.2
Flank skinfold thickness*	592	0.032 (-0.042—0.105)	0.4	0.017 (-0.056—0.089)	0.7
Quadriceps skinfold thickness*	592	0.050 (-0.013—0.112)	0.1	0.042 (-0.020—0.105)	0.2
Subscapular skinfold thickness*	592	0.069 (-0.001—0.139)	0.05	0.061 (-0.009—0.132)	0.09
Triceps skinfold thickness*	592	0.062 (-0.009—0.133)	0.09	0.056 (-0.015—0.126)	0.1

Values in the table only include data from those pregnancies where GDM status was available. Standardized βs are presented as means (95% confidence intervals). All models adjusted for gestational age at birth, parity, smoking during pregnancy, offspring sex and maternal pre-pregnancy BMI. *Models additionally adjusted for age at assessment.

Table S6 Subgroup comparison assessing the effect of maternal weight gain in pregnancy on associations between iron supplementation in pregnancy and offspring size at birth.

Measure	N	Unadjusted for maternal weight gain		Adjusted for maternal weight gain	
		Standardized β	p-value	Standardized β	p-value
Weight	632	0.075 (0.012—0.137)	0.02	0.067 (0.005—0.128)	0.03
Length*	619	0.038 (-0.020—0.095)	0.2	0.031 (-0.025—0.088)	0.3
Head Circumference*	618	0.089 (0.033—0.144)	0.002	0.084 (0.029—0.139)	0.003
BMI*	618	0.078 (0.017—0.138)	0.01	0.074 (0.013—0.134)	0.02
Ponderal Index*	618	0.061 (0.002—0.120)	0.04	0.059 (0—0.119)	0.05
Flank skinfold thickness*	617	0.082 (0.012—0.151)	0.02	0.080 (0.010—0.150)	0.02
Quadriceps skinfold thickness*	617	0.059 (-0.003—0.121)	0.06	0.056 (-0.006—0.118)	0.08
Subscapular skinfold thickness*	618	0.105 (0.036—0.174)	0.003	0.100 (0.031—0.169)	0.005
Triceps skinfold thickness*	617	0.120 (0.047—0.193)	0.001	0.116 (0.043—0.189)	0.002

Values in the table only include data from those pregnancies where maternal weight gain in pregnancy was available. Standardized βs are presented as means (95% confidence intervals). All models adjusted for gestational age at birth, parity, smoking during pregnancy, offspring sex and maternal pre-pregnancy BMI. *Models additionally adjusted for age at assessment.
